# Physical Activity and Risk of Diagnosed and Undiagnosed Prediabetes among Males and Females in the National Health and Nutrition Examination Survey, 2007-2014

**DOI:** 10.1155/2020/3538746

**Published:** 2020-06-11

**Authors:** Mabeline Velez, Lisa Chasan-Taber, Eva Goldwater, Nicole VanKim

**Affiliations:** Department of Biostatistics and Epidemiology, School of Public Health and Health Sciences, University of Massachusetts Amherst, Amherst Massachusets, USA

## Abstract

**Aims:**

The purpose of the study was to assess the effect of leisure and occupational physical activity on the risk of diagnosed and undiagnosed prediabetes among females and males.

**Methods:**

A sample of 17,871 non-pregnant adults was drawn from the 2007-2014 National Health and Nutrition Examination Survey. Multinomial logistic regression tested associations between moderate-to-vigorous physical activity (MVPA) and risk of diagnosed prediabetes and undiagnosed prediabetes, compared to no prediabetes.

**Results:**

Females and males who met guidelines for total MVPA (i.e., ≥10 MET-hrs/week) had a statistically significant lower risk of undiagnosed prediabetes (OR range: 0.50-0.65) as compared to those with no MVPA, however findings were no longer statistically significant after adjustment for diabetes risk factors. In terms of diagnosed prediabetes, females meeting guidelines had lower risk (OR range: 0.65-0.76), while only males engaging in the most MVPA had lower risk; findings were no longer significant after adjustment. Patterns were similar for leisure-time MVPA, but conflicting for occupational PA; females with 10-20 MET-hrs/week had a higher risk of diagnosed prediabetes (OR =1.71, 95% CI 1.11-2.61) and males with >20 MET-hrs/week had a higher risk for undiagnosed prediabetes (OR =1.17, 95% CI 1.02-1.35) after adjustment.

**Conclusions:**

This study adds to the sparse body of literature on physical activity and prediabetes, particularly with its inclusion of occupational MVPA.

## 1. Introduction

Prediabetes is a serious health condition in which glucose levels are above normal but not high enough to be classified as type 2 diabetes [1]. Specifically, the term “prediabetes” is used to refer to individuals with impaired fasting glucose (IFG) and/or impaired glucose tolerance (IGT) and indicates an increased risk for the future development of diabetes [2]. According to the Centers for Disease and Prevention's 2017 National Diabetes Statistics Report, 84.1 million adults, or approximately 1 in 3 had prediabetes in 2015 with higher prevalence in males than in females (i.e., 36.6% vs. 29.3%) [1]. Despite this high prevalence, only 11.6% of prediabetes cases were aware that they had this condition [1]. This is critical as without weight loss (when indicated), healthy eating, and moderate physical activity, individuals with prediabetes have an increased risk of developing subsequent type 2 diabetes. Indeed, recent studies have found that individuals who have prediabetes have a 20 times higher risk of progressing to type 2 diabetes within five years of diagnosis as compared to those with normal glucose levels [3].

Physical activity is important in regulating glucose levels among individuals who have diabetes, and therefore, the American Diabetes Association (ADA) recommends that adults engage in a total of 150 minutes of weekly moderate to vigorous physical activity [2, 4]. A meta-analysis of physical activity and type 2 diabetes found evidence for an inverse association between all subtypes of physical activity (e.g., leisure-time and occupational) and risk of type 2 diabetes with somewhat larger reductions in risk observed for leisure-time activity [5]. However, the impact of physical activity, as well as specific subtype of physical activity, on preventing prediabetes is less clear. Specifically, prior studies on the association between leisure-time physical activity and prediabetes are sparse [6–12], and only one study, to our knowledge, evaluated the association between occupational physical activity and prediabetes [12].

Prior studies evaluating the impact of sex on risk of type 2 diabetes found that physical activity was separately and significantly associated with lower risk of diabetes among both men and women [13, 14]. However, in terms of prediabetes, the majority of prior studies did not evaluate findings for males and females separately. This is critical as emerging studies suggest that there are sex differences in glucose and lipid metabolism and possibly an increased metabolic flexibility in females; with observations of higher insulin stimulated glucose uptake in female skeletal muscle despite greater body fat stores and greater lipid stores in the skeletal muscle of females than in males [15].

Finally, prior studies did not distinguish between diagnosed prediabetes and undiagnosed prediabetes. This is important as those who receive a diagnosis of prediabetes may be advised to increase their physical activity level therefore making it more difficult to elucidate the temporal sequence between leisure-time activity and diagnosed prediabetes as compared to undiagnosed prediabetes.

Therefore, we investigated the association between physical activity and risk of diagnosed and undiagnosed prediabetes among females and males in the National Health and Nutrition Examination Survey (NHANES) from the years 2007-2014. We hypothesized that 1) there would be an inverse relationship between physical activity (both leisure-time and occupational) and prediabetes, and 2) the effect of physical activity (both leisure-time and occupational) on prediabetes would be stronger in females compared to males.

## 2. Methods

### 2.1. Study Population

We used data from the 2007-2014 NHANES, a complex, multistage probability survey that examines a nationally representative sample of the US population of all ages [16]. Participants were interviewed at home followed by a clinical examination in a mobile examination center. The Centers for Disease Control and Prevention/National Center for Health Statistics (CDC/NCHS) Ethics Review Board (ERB) approved the study.

A total of 40,617 individuals participated in the NHANES 2007-2014. For the purposes of the current analysis, we limited inclusion to individuals with data available on prediabetes status, borderline diabetes status, or hemoglobin A1c (HbA1c) laboratory data. We excluded individuals with: 1) self-reported diabetes, 2) HbA1c levels above 6.5% (48 mmol/mol), 3) a history of coronary heart disease, heart failure, angina pectoris, heart attack, 4) females who were pregnant at time of examination, and 5) individuals younger than 18 years of age at time of examination.

### 2.2. Prediabetes Assessment

Prediabetes was defined according to the HbA1c criteria of the ADA [2]. Specifically, the Diabetes Interview Questionnaires (DIQ) [17] and HbA1c biomarkers were used to categorize individuals into three categories: diagnosed prediabetes, undiagnosed prediabetes, and normal. Individuals who answered “yes” to the question, “Have you ever been told you had prediabetes?” or “borderline” to the question, “Have you ever been told by a doctor or health professional that you have diabetes or sugar diabetes?” were classified as having diagnosed prediabetes. Individuals who answered “no” to the above questions but had a HbA1c level between 5.7% (39 mmol/mol) and 6.4% (46 mmol/mol) were classified as having undiagnosed prediabetes. Those who answered “no” to the above questions and had HbA1c levels below 5.7% (39 mmol/mol) were classified as having normal glucose levels.

In 2015, the National Glycohemoglobin Standardization Program (NGSP) reviewed the NHANES laboratory and participant HbA1c data and concluded that the NHANES laboratories met the NGSP criteria for bias and precision [2].

### 2.3. Physical Activity Assessment

Physical activity was assessed by the Global Physical Activity Questionnaire (GPAQ), [18] a validated tool which assesses the total time spent in leisure-time, occupational, and travel domains of activity in a typical week. Leisure-time activity included moderate and vigorous sports, fitness, or recreational activities. Occupational physical activity included paid or unpaid work, studying or training, and household chores. Travel physical activity included walking or bicycling for transportation. Weekly hours of occupational and leisure activities were recorded separately for vigorous and moderate levels of physical activity intensity.

Weekly hours of moderate-intensity activity in occupational and leisure activities and walking/bicycling for transportation were multiplied by the metabolic equivalent of task (MET) of 4 to derive MET-hours/week in moderate-intensity activity within each domain of activity. Similarly, weekly hours of vigorous-intensity activity were multiplied by 8 METs to derive MET-hours/week in vigorous activity within each domain of activity. We then summed moderate and vigorous MET-hours/week to derive occupational moderate-to-vigorous physical activity (MVPA) and leisure MVPA, respectively. In addition, we created a total MVPA variable by summing occupational, leisure and transportation weekly MET-hours per week [19, 20].

Each physical activity variable was categorized into four levels based on the US Department of Health and Human Services: 1) none, 2) <10 MET-hours/week, 3) 10-20 MET- hours/week, and 4) >20 hours/week [21]. The latter two categories (i.e., ≥10 MET-hours/week) are equivalent to meeting or exceeding the current PA recommendations of 150 minutes or more of MVPA per week [21].

### 2.4. Assessment of Covariates

Information on age, race/ethnicity, education, and the family/poverty income ratio were collected via the demographic questionnaire. The family poverty/income ratio, a ratio of poverty income to the federal poverty threshold, was categorized into three groups: poor (≤1.3), near poor (1.3-3.5), and non-poor (≥3.5). Smoking status was categorized into never, past smoker and current smoker, based on the smoking questionnaire. Body mass index (BMI) was based on weight (kg) and height (m) and categorized as underweight (<18.5 kg/m^2^), normal (18.5 to <25 kg/m^2^), overweight (25 to <30 kg/m^2^) and obese (≥30 kg/m^2^). Hypertension was defined as having received a diagnosis of hypertension, mean systolic blood pressure ≥140 mmHg or mean diastolic blood pressure ≥90 mmHg, or having been advised to take medication for hypertension. Family history of diabetes was assessed via self-report. Diet quality was assessed using the Health Eating Index (HEI), a diet quality index that measures alignment with the Dietary Guidelines for Americans [22].

### 2.5. Statistical Analysis

Descriptive statistics were stratified by sex and included the calculation of means and standard deviation for continuous variables, and frequencies and percentages for categorical variables. Chi-square tests were performed to analyze bivariate associations between the covariates and prediabetes status, stratified by sex.

We used multinomial logistic regression to analyze the association between the physical activity variables and prediabetes status (i.e., undiagnosed prediabetes, diagnosed prediabetes) stratifying by sex. Model 1 included unadjusted results. For Model 2, we used backwards elimination with a p value of 0.1 as a cutoff value for inclusion. Using this criteria, age, race/ethnicity, body mass index, education, smoking status, family history of diabetes, and hypertension were included in adjusted models; while poverty/income ratio and HEI scores were eliminated. Model 3 additionally included the other domain of MVPA; for example when evaluating the association between leisure-time MVPA and prediabetes, we adjusted for occupational MVPA. Tests for trend were conducted by including the categorical PA variables as continuous variables in the regression model (e.g., 0, 1, 2, 3) and calculating the corresponding p value. All analyses applied the corresponding NHANES survey weights. Statistical analyses were conducted using STATA version 16.0.

## 3. Results

A total of 40,617 individuals participated in NHANES 2007-2014. After excluding individuals with self-reported diabetes (n =903); HbA1c levels above 6.5% (48 mmol/mol) (n = 1,972); a history of coronary heart disease, heart failure, angina pectoris, or heart attack (n = 1,981); pregnant at time of examination (n = 540); younger than 18 years of age at time of examination (n = 15,835); and those missing information on diabetes status (n = 1,515); 17,871 individuals (9,134 females and 8,737 males) were included in the final analytic sample.

The average age of the study population was 43 years and the sample was predominantly non-Hispanic white (68%); 14% of the sample was Hispanic and 11% Black (Table 1). Over two-thirds of the sample were overweight or obese (66%) with higher prevalence of overweight/obesity in males (69%) compared to females (61%, p < 0.0001). Males were also more likely to be current smokers than females (24% vs. 18%, p < 0.0001).

In terms of physical activity, 67% of the sample met guidelines for total MVPA (i.e., 10 or more MET-hrs/wk) with lower prevalence in females (59.6%) than in males (75.4%, p < 0.0001) (Table 1). Similar patterns were observed for leisure-time and occupational MVPA with prevalence of physical activity consistently lower in females than in males. For example, only 28.6% of females engaged in at least 10 MET-hrs/wk of occupational MVPA, compared to 44.7% of males (p < 0.0001).

Overall, 1,084 individuals (6.0%) were diagnosed with prediabetes, 4,300 (24.1%) had undiagnosed prediabetes, and 12,487 (69.9%) had normal glucose levels. We then assessed the bivariate association between covariates and prediabetes status among females and males, respectively (Table 2). Those with undiagnosed and diagnosed prediabetes were more likely to be obese, have lower levels of education, have a family history of diabetes, and have hypertension than those with normal glucose levels. HEI scores did not differ significantly according to prediabetes status.

We then evaluated the association between physical activity and prediabetes in females (Table 3). In unadjusted models, there was a statistically significant decreasing trend in risk for undiagnosed prediabetes for each increasing level of total MVPA (p_trend_< 0.0001) among females. More specifically, females meeting guidelines for total MVPA (i.e., 10-20 MET-hrs/wk or> 20 MET-hrs/wk) had a statistically significant lower risk of undiagnosed prediabetes (OR range: 0.51-0.62) and diagnosed prediabetes (OR range: 0.65-0.76) as compared to those reporting no MVPA. However, after adjusting for important diabetes risk factors such as age, BMI, race/ethnicity, education, smoking status, family history of diabetes, and hypertension, these associations were attenuated and no longer statistically significant.

Findings were similar for leisure-time MVPA in terms of overall trend in the association between increasing levels of physical activity and risk of undiagnosed and diagnosed prediabetes (p_trend_< 0.001) (Table 3). Specifically, females with >20 MET-hrs/wk of leisure-time MVPA had a lower risk of undiagnosed prediabetes (OR = 0.46, 95% CI 0.38-0.56) and diagnosed prediabetes (OR = 0.58, 95% CI 0.43-0.78) as compared to those reporting no leisure-time MVPA. However, again findings were attenuated and no longer statistically significant after covariate adjustment. Findings were somewhat more modest for occupational activity with a less consistent, but still statistically significant decreasing trend in risk of undiagnosed prediabetes (p < 0.0001) and smaller reductions in risk of undiagnosed prediabetes for those with >20 MET-hrs/wk (OR = 0.72, 95% CI 0.61-0.85). Again, the findings were attenuated upon adjustment. Findings for risk of diagnosed prediabetes suggested that females who engaged in more occupational MVPA had higher risk than those who did not engage in occupational MVPA, with those engaging in 10-20 MET-hrs/wk, having a statistically significantly higher risk (OR =1.71, 95% CI 1.11-2.61), after adjusting for risk factors.

We then evaluated the association between physical activity and prediabetes in males (Table 4). In unadjusted models, males meeting guidelines for total MVPA had a statistically significant lower risk of undiagnosed prediabetes (OR range: 0.55-0.65) which was attenuated and no longer statistically significant after adjustment for important risk factors. Only males with the highest levels of total MVPA (>20 MET-hrs/week) had a lower risk of diagnosed prediabetes (OR = 0.51, 95% CI 0.38-0.69, p_trend_< 0.0001) as compared to those reporting no MVPA, but this association was no longer significant after adjustment.

Findings were similar for leisure-time MVPA (Table 4); males meeting guidelines for leisure-time MVPA had a lower risk of undiagnosed prediabetes (OR range: 0.46-0.67). For diagnosed prediabetes, only males with >20 MET-hrs/week had a statistically lower risk (OR = 0.55, 95% CI 0.41-0.74) as compared to those reporting no leisure-time MVPA. However, again findings were typically attenuated and no longer statistically significant after adjustment. Findings were conflicting for occupational activity. Among males with the highest levels of occupational activity (>20 MET-hrs/week), we observed no difference in risk for undiagnosed prediabetes, however, after adjustment for important risk factors, there was a higher risk for undiagnosed prediabetes (OR =1.17, 95% CI 1.02-1.35, p_trend_ = 0.018). There was a lower risk of diagnosed prediabetes (OR = 0.72, 95% CI 0.54-0.96, p_trend_ = 0.043), which was no longer statistically significant after adjustment.

## 4. Discussion

In this large cross-sectional study using national data, we found that females and males who met guidelines for total MVPA (i.e., at least 10 MET-hrs/week) had statistically significantly lower risk of undiagnosed prediabetes. We also found that there was generally a statistically significant decreasing trend in risk for undiagnosed prediabetes with increasing levels of total MVPA. However, these findings were attenuated and no longer statistically significant after adjustment for important diabetes risk factors. In terms of diagnosed prediabetes, females meeting total MVPA guidelines had a lower risk, while only men with the highest total MVPA experienced lower risk; again findings were no longer significant after adjustment. Similar patterns were observed for leisure-time MVPA in both males and females. However, findings were conflicting for occupational MVPA, with the suggestion of a higher risk of undiagnosed prediabetes, but not diagnosed prediabetes, among males with the highest levels of occupational physical activity.

Our findings for total MVPA are consistent with the majority of prior studies which evaluated total MVPA [7, 9–11] and found, with only one exception [11], that observations of a protective effect for prediabetes were attenuated and no longer statistically significant after adjustment for diabetes risk factors. For example, Farni et al. examined the relationship between total MVPA as measured by accelerometry and prediabetes among participants in NHANES from 2003-2006 [10]. The authors found that after adjusting for BMI, those in the highest tertile were 0.77 times as likely to have prediabetes as BMI-matched controls in the lowest tertile; however this effect was attenuated and no longer significant upon further adjustment for age. Similarly, in a study in Ellisras, South Africa, Matshipi et al. found that there was no association between total physical activity as measured by the International Physical Activity Questionnaire and prediabetes after adjustment for covariates [7].

Our findings for leisure-time MVPA are consistent with some [6], but not all [8, 12] of the few previous studies which evaluated this domain of physical activity and risk of prediabetes. For example, in the Mexican Health and Aging Study, Kumar et al. found that those engaging in higher levels of physical activity (defined as vigorous activity or exercise three times a week or more) did not have a significant reduction in odds of prediabetes [6]. Wang et al. using NHANES data from 2007–2012 found that high levels of total leisure-time physical activity (OR = 0.78, 95% CI 0.66-0.94) and low levels of vigorous leisure-time physical activity (OR = 0.72, 95% CI 0.58-0.90) were inversely associated with the risk of prediabetes in multivariate adjusted models [8]. However, analyses were not conducted separately for undiagnosed and diagnosed prediabetes nor for males and females.

Only one prior study, to our knowledge, evaluated the association between occupational activity and risk of prediabetes. In the Midlife in the United States (MIDUS) study conducted from 1995-2006, Tsenkova et al. found a nonsignificant increase in risk between self-reported occupational physical activity and prediabetes (*β* = 0.08, 95% CI −0.10 - 0.26) after adjusting for diabetes risk factors and other forms of physical activity [12]. However, the authors did not evaluate associations separately for males and females, nor for diagnosed and undiagnosed prediabetes.

By way of comparison with risk of type 2 diabetes, a recent meta-analysis found that while most studies reported reduced risk with greater leisure-time activity or vigorous activity, data were less consistent for studies investigating moderate intensity activity with some reporting an inverse association and others finding no significant association with type 2 diabetes [5]. While leisure-time activities were associated with a 25–40% reduction in the relative risk of type 2 diabetes, occupational activity was associated with a 15% decrease in risk [5].

Notably, our finding that engaging in occupational physical activity may be associated with a higher risk of prediabetes adds to the emerging literature that questions the health benefits of occupational physical activity. For example, while some prior studies have found no significant associations between occupational physical activity and cardiovascular risk factors [23, 24], more recent prior studies have observed positive associations with obesity and insulin resistance [25]. It has been proposed that the heavy lifting, prolonged standing, and highly repetitive work that is characteristic of occupational MVPA may not have the same beneficial impact on glucose regulation as that observed for the aerobic and resistance training characteristics of leisure-time MVPA [4, 12]. This finding of an increased risk of prediabetes for occupational MVPA could also be due to confounding by irregular working hours, stress, and other adverse factors associated with occupational activity.

Overall, we observed similar findings for males and females. Although emerging studies suggest that there are sex differences in glucose and lipid metabolism and possibly an increased metabolic flexibility in females [15], there is a paucity of research examining the effects of exercise on health in women and some studies suggest that there may be a discordance in the effectiveness of exercise regimens between the sexes. For example, while high intensity interval training has been observed to increase aerobic capacity in both men and women, its ability to enhance insulin sensitivity appears to be blunted in women, which may have implications for prediabetes and diabetes but remains to be studied [26]. While prior studies evaluating the impact of sex on risk of type 2 diabetes found that physical activity was separately and significantly associated with lower risk of diabetes among both men and women, such studies are sparse [13, 14].

Our study has several strengths including the large and representative sample which enabled us to evaluate associations separately by sex, by diagnosis status, and by specific domain of physical activity. We also had the ability to adjust for a wide range of important risk factors including the other domain of activity in each analysis. Finally, this study adds to the sparse literature on physical activity and prediabetes, particularly with its inclusion of occupational MVPA.

Our study had several limitations. The cross-sectional study design limits the establishment of the causal associations between physical activity and the risk of prediabetes as individuals who receive a diagnosis of prediabetes may seek to increase their physical activity level as a form of treatment. However, our analysis of undiagnosed prediabetes would not face this concern. Secondly, information on physical activity was obtained by self-report, however, this concern is reduced by the use of a validated questionnaire. Third, the condition of ‘prediabetes' is more accurately described by IGF and IGT although the NHANES dataset does not distinguish between these conditions. However, as noted by the American Diabetes Association, IFG and IGT should not be viewed as clinical entities in their own right but rather risk factors for diabetes [2]. Our reliance upon both self-reported diagnosis of prediabetes in conjunction with laboratory results was a study strength.

In conclusion, consistent with other studies using NHANES data, we found that while 6.0% of our participants were diagnosed with prediabetes, almost one-quarter (24.1%) had undiagnosed prediabetes [27–29]. The Centers for Disease Control and Prevention estimates that 84 million people, at least a third of the nation's adult population, are living with prediabetes while 90% of those with prediabetes are not aware of their condition [30]. This highlights the need for early detection of and regular screening for prediabetes such that those with undiagnosed prediabetes can take advantage of prevention strategies. Indeed, there is strong evidence to support the ability of lifestyle modifications including exercise in delaying the progression from prediabetes to type 2 diabetes [31]. In addition, future research studies including women are needed to determine the mechanisms that underlie, and the impact of, exercise on the health of women. Finally, the findings from the current study suggest that counseling and other interventions focused on increasing leisure-time physical activity among those with diagnosed prediabetes should be targeted.

## Figures and Tables

**Table 1:** Descriptive Characteristics^∗^ according to Sex; NHANES, 2007-2014, (N = 17,871).

Characteristics	Overall (n = 17,871)	Female (n = 9,134)	Male (n = 8,737)	*p value*
	n (%)	n (%)	n (%)	
Age (mean, SD)	43.48 (16.68)	44.81 (17.09)	42.06 (16.00)	*< 0.0001*
Race/ethncity				
White	7,781 (67.89)	4,012 (68.44)	3,769 (67.29)	*<0.0001*
Black	3,659 (10.83)	1,854 (11.39)	1,805 (10.23)	
Hispanic	4,553 (14.25)	2,344 (13.3)	2,209 (15.27)	
Other	1,878 (7.03)	924 (6.87)	954 (7.21)	
Body mass index				*<0.0001*
Underweight (<18.5 kg/m^2^)	386 (1.95)	243 (2.52)	143 (1.33)	
Normal (18.5-<25 kg/m^2^)	5,624 (33.33)	2,974 (36.40)	2,650 (30.10)	
Overweight (25-<30 kg/m^2^)	5,669 (33.84)	2,522 (28.86)	3,147 (39.17)	
Obese (≥30 kg/m^2^)	5,439 (31.88)	3,075 (32.22)	2,364 (29.44)	
Education				*<0.0001*
Less than high school	4,419 (16.89)	2,137 (15.95)	2,282 (17.89)	
High school graduate	4,178 (22.51)	2,009 (21.16)	2,169 (23.96)	
Some college	5,216 (31.22)	2,892 (33.39)	2,324 (28.88)	
College graduate or higher	4,037 (29.38)	2,085 (29.50)	1,952 (29.27)	
Family poverty/income ratio				*<0.0001*
≤1.3	5,515(23.01)	2,957 (24.3)	2,558 (21.63)	
1.3-3.5	5,764 (34.34)	2,932 (34.84)	2,832 (33.81)	
> 3.5	4, 998 (42.65)	2,449 (40.86)	2,549 (44.56)	
Smoking status				*<0.0001*
Never	9,755 (57.15)	5,688 (62.22)	4,067 (51.68)	
Past	3,558 (21.81)	1,477 (19.35)	2,081 (24.47)	
Current	3,700 (21.04)	1,568 (18.43)	2, 132 (23.85)	
Family history of diabetes				*<0.0001*
Yes	5,823 (33.39)	3,198 (35.36)	2,625 (31.26)	
No	10,554 (66.61)	5,208 (64.64)	5,346 (68.74)	
Hypertension				*0.6*
Yes	5,783 (29.69)	3,000 (29.90)	2,783 (29.47)	
No	12,085 (70.31)	6,132 (70.10)	5,953 (29.47)	
Healthy eating index score (mean, SD)	55.13 (13.36)	56.19 (13.18)	54.01 (13.19)	*<0.0001*
Total MVPA				
None	4,173 (19.72)	2,615 (24.46)	1,558 (14.66)	*<0.0001*
<10 MET-hrs/wk	2,371 (13.07)	1,466 (15.97)	905 (9.98)	
10-20 MET-hrs/wk	2,237 (12.98)	1,323 (15.15)	914 (10.66)	
>20 MET-hrs/wk	9.090 (54.22)	3,730 (44.43)	5,360 (64.71)	
Leisure-time MVPA				*<0.0001*
None	8,745 (43.33)	4,820 (46.39)	3,925 (40.05)	
<10 MET-hrs/wk	2,663 (16.45)	1,498 (17.92)	1,165 (14.88)	
10-20 MET-hrs/wk	2,330 (14.81))	1,213 (15.31)	1,117 (14.27)	
>20 MET-hrs/wk	4,133 (25.42)	1,603 (20.39)	2,530 (30.80)	
Occupational MVPA				*<0.0001*
None	10,598 (56.07)	6,076 (63.43)	4,522 (48.21)	
<10 MET-hrs/wk	1,225 (7.54)	655 (7.96)	570 (7.09)	
10-20 MET-hrs/wk	1,046 (6.68)	506 (6.43)	540 (6.95)	
>20 MET-hrs/wk	5,002 (29.71)	1,897 (22.18)	3,105 (37.75)	

Abbreviations: MET, Metabolic Equivelent; MVPA = moderate-to-vigorous physical activity. ∗Percents, means, and standard deviations are weighted; counts are sampled number of observations; numbers may not sum to 17,871 due to missing data.

**Table 2:** Bivariate Associations^∗^ between Covariates and Prediabetes Status; NHANES, 2007-2014, (N = 17,871.

Characteristics	Female				Male			
	Normal (N = 6,283)	Undiagnosed prediabetes (N = 2,214)	Diagnosed prediabetes (N = 637)	*p value*	Normal (N = 6,204)	Undiagnosed prediabetes (N = 2,086)	Diagnosed prediabetes (N = 447)	*p value*
	n (%)	n (%)	n (%)		n (%)	n (%)	n (%)	
Age (mean, SD)	40.77 (15.26)	56.38 (16.73)	51.57 (15.91)	*<0.0001*	38.93 (14.57)	50.99 (17.19)	53.69 (13.21)	*<0.0001*
Race/ethncity				*0.0001*				*<0.0001*
White	2,853 (69.59)	874 (64.32)	285 (69.05)		2,854 (69.65)	695 (56.41)	220 (74.0)	
Black	1,160 (10.14)	556 (15.71)	138 (11.24)		1,106 (8.52)	614 (17.41)	85 (7.97)	
Hispanic	1,618 (13.51)	578 (13.13)	148 (11.56)		1,556 (15.01)	555(17.34)	98(11.25)	
Other	652 (6.75)	206 (6.84)	66 (8.15)		688 (6.81)	222(8.85)	44 (6.78)	
Body mass index				*<0.0001*				*<0.0001*
Underweight (<18.5 kg/m^2^)	203 (2.98)	32 (1.45)	8 (1.07)		118 (1.52)	22 (0.95)	3 (0.38)	
Normal (18.5-<25 kg/m^2^)	2,368 (41.44)	490 (24.64)	116 (19.86)		2,136 (33.77)	434 (19.17)	80 (16.98)	
Overweight (25-<30 kg/m^2^)	1,695 (28.48)	669 (30.97)	158 (26.32)		2,165 (39.15)	807 (38.41)	175 (42.30)	
Obese (≥30 kg/m^2^)	1,733 (27.51)	1,000 (42.95)	342 (52.76)		1,397(25.58)	800 (41.49)	167 (40.34)	
Education				*<0.0001*				*<0.0001*
Less than high school	1,347 (14.47)	648 (21.05)	142 (15.77)		1,508 (16.19)	667 (24.56)	107 (17.46)	
High school graduate	1,353 (19.85)	521 (25.81)	1325 (20.55)		1,509 (23.21)	549 (26.82)	111 (24.09)	
Some college	2,035 (34.11)	635 (29.72)	222 (37.14)		1,726 (29.74)	485 (26.63)	113 (25.01)	
College graduate or higher	1,543 (31.56)	405 (23.42)	137 (26.54)		1,452 (30.86)	384 (22.00)	116 (33.43)	
Family poverty/income ratio				*0.0013*				*0.0422*
≤1.3	2,072 (24.5)	680 (24.36)	205 (22.09)		1,850 (21.7)	599 (22.98)	109 (15.7)	
1.3-3.5	1,944 (32.95)	775 (40.58)	213 (37.36)		1,977 (33.54)	704 (35.28)	151 (32.3)	
> 3.5	1,739 (42.55)	532 (35.06)	178 (40.55)		1,829 (44.76)	566 (41.73)	154 (52.00)	
Smoking status				*0.016*				*<0.0001*
Never	3,928 (63.35)	1,372 (59.39)	388 (59.58)		3,004 (54.13)	884 (44.66)	179 (43.82)	
Past	916 (17.93)	427 (21.90)	134 (25.88)		1,282 (22.55)	608 (27.27)	191 (40.50)	
Current	1,082 (18.72)	381 (18.74)	105 (14.54)		1,503 (23.31)	552 (28.07)	77 (15.68)	
Family history of diabetes				*<0.0001*				*<0.0001*
Yes	1,982 (32.36)	889 (40.66)	327 (49.2)		1,674 (28.46)	721 (35.92)	230 (51.78)	
No	3,680 (67.64)	1,244 (59.34)	284 (50.8)		3,863 (71.54)	1,275 (64.08)	208 (48.22)	
Hypertension				*<0.0001*				*<0.0001*
Yes	1,533 (22.56)	1,102 (46.55)	365 (55.64)		1,574 (24.16)	944 (42.95)	265 (55.41)	
No	4,748 (77.44)	1,112 (53.45)	272 (44.36)		4,629 (75.84)	1,142 (57.05)	182 (44.59)	
Healthy eating index score (mean, SD)	56.07 (12.82)	56.50 (14.19)	56.41 (13.68)	*0.49*	53.93 (12.89)	53.73 (14.17)	56.09 (12.62)	*0.156*
Total MVPA				*<0.0001*				*<0.0001*
None	1,658 (21.91)	772 (31.94)	185 (28.19)		971 (12.80)	482 (20.23)	105 (20.56)	
<10 MET-hrs/wk	975 (15.1)	379 (18.58)	112 (17.02)		616 (9.74)	237 (10.96)	52 (9.80)	
10-20 MET-hrs/wk	909 (15.5)	306 (13.96)	108 (15.12)		636 (10.39)	219 (10.63)	59 (14.57)	
>20 MET-hrs/wk	2,741 (47.49)	757 (35.52)	232 (39.67)		3,981 (67.07)	1,148 (58.18)	231 (55.06)	
Leisure-time MVPA				*<0.0001*				*<0.0001*
None	3,164 (43.5)	1,307 (54.83)	349 (50.67)		2,538 (36.54)	1,160 (52.46)	227 (44.22)	
<10 MET-hrs/wk	1,024 (17.59)	360 (18.22)	114 (20.33)		830 (14.81)	267 (14.27)	68 (18.1)	
10-20 MET-hrs/wk	858 (15.99)	272 (13.58)	83 (13.48)		834 (15.05)	216 (11.00)	67 (15.33)	
>20 MET-hrs/wk	1,237 (22.91)	275 (13.37)	91 (15.52)		2,002 (33.6)	443 (22.27)	85 (22.35)	
Occupational MVPA				*0.001*				*0.13*
None	4,110 (62.32)	1,562 (68.48)	404 (59.58)		3,186 (47.97)	1,097 (47.99)	239 (52.4)	
<10 MET-hrs/wk	443 (7.76)	154 (8.21)	58 (9.24)		404 (7.02)	128 (6.74)	38 (9.30)	
10-20 MET-hrs/wk	363 (6.65)	98 (4.92)	45 (8.73)		365 (6.75)	143 (7.4)	32 (8.16)	
>20 MET-hrs/wk	1,367 (23.27)	400 (18.39)	130 (22.45)		2,249 (38.26)	718 (37.86)	138 (30.14)	

Abbreviations: MET, Metabolic Equivelent; MVPA = moderate-to-vigorous physical activity. ∗Percents, means, and standard deviations are weighted; counts are sampled number of observations; numbers may not sum to 17,871 due to missing data.

**Table 3:** Odds ratios and 95% Confidence Intervals for the Association between Physical Activity and Prediabetes among Females; NHANES, 2007-2014.

	Total sample	Undiagnosed prediabetes										Diagnosed prediabetes									
		Cases	Model 1^∗^			Model 2^†^			Model 3^‡^			Cases	Model 1^∗^			Model 2^†^			Model 3^‡^		
			OR	95% confidence interval		OR	95% confidence interval		OR	95% confidence interval			OR	95% confidence interval		OR	95% confidence interval		OR	95% confidence interval	
Total MVPA																					
None	2,615	772	1.00	Referent		1.00	Referent		n/a	n/a	n/a	185	1.00	Referent		1.00	Referent		n/a	n/a	n/a
<10 MET-hrs/wk	1,466	379	0.84	0.72	1.00	1.23	1.03	1.47	n/a	n/a	n/a	112	0.88	0.63	1.23	1.12	0.77	1.63	n/a	n/a	n/a
10-20 MET-hrs/wk	1,323	306	0.62	0.51	0.75	1.05	0.84	1.31	n/a	n/a	n/a	108	0.76	0.58	1.00	1.17	0.87	1.57	n/a	n/a	n/a
>20 MET-hrs/wk	3,730	757	0.51	0.43	0.61	1.03	0.84	1.26	n/a	n/a	n/a	232	0.65	0.50	0.84	1.20	0.93	1.53	n/a	n/a	n/a
*p trend*					*<0.0001*			*0.888*							*0.001*			*0.199*			
Leisure-time MVPA																					
None	4,820	1,307	1.00	Referent		1.00	Referent		1.00	Referent		349	1.00	Referent		1.00	Referent		1.00	Referent	
<10 MET-hrs/wk	1,498	360	0.82	0.71	0.95	1.22	1.02	1.44	1.22	1.03	1.45	114	0.99	0.77	1.27	1.31	1.01	1.70	1.29	1.00	1.67
10-20 MET-hrs/wk	1,213	272	0.67	0.54	0.83	1.07	0.83	1.39	1.08	0.83	1.40	83	0.72	0.50	1.06	1.09	0.73	1.61	1.06	0.72	1.55
>20 MET-hrs/wk	1,603	275	0.46	0.38	0.56	0.95	0.74	1.21	0.96	0.75	1.23	91	0.58	0.43	0.78	1.09	0.80	1.50	1.06	0.77	1.46
*p trend*					*<0.0001*			*0.886*			*0.963*				*0.001*			*0.566*			*0.737*
Occupational MVPA																					
None	6,076	1,562	1.00	Referent		1.00	Referent		1.00	Referent		404	1.00	Referent		1.00	Referent		1.00	Referent	
<10 MET-hrs/wk	655	154	0.96	0.78	1.19	1.08	0.84	1.39	1.07	0.83	1.37	58	1.25	0.89	1.74	1.27	0.91	1.78	1.25	0.89	1.76
10-20 MET-hrs/wk	506	98	0.67	0.48	0.94	0.88	0.62	1.24	0.88	0.61	1.25	45	1.37	0.90	2.09	1.71	1.12	2.63	1.71	1.11	2.61
>20 MET-hrs/wk	1897	400	0.72	0.61	0.85	0.92	0.76	1.11	0.92	0.75	1.11	130	1.01	0.80	1.28	1.24	0.97	1.59	1.24	0.97	1.58
*p trend*					*<0.0001*			*0.327*			*0.324*				*0.599*			*0.024*			*0.025*

Abbreviations: MET, Metabolic Equivelent; OR, Odds Ratio. ^∗^Model 1: Unadjusted. ^†^Model 2: Adjusted for age, race/ethncity, body mass index, education, smoking status, family history of diabetes, hypertension. ^‡^Model 3: Adjusted for age, race/ethncity, body mass index, education, smoking status, family history of diabetes, hypertension, and leisure-time or occupational MVPA.

**Table 4:** Odds ratios and 95% Confidence Intervals for the Association between Physical Activity and Prediabetes among Males; NHANES, 2007-2014.

	Total sample	Undiagnosed prediabetes										Diagnosed prediabetes									
		Cases	Model 1^∗^			Model 2^†^			Model 3^‡^			Cases	Model 1^∗^			Model 2^†^			Model 3^‡^		
			OR	95% confidence interval		OR	95% confidence interval		OR	95% confidence interval			OR	95% confidence interval		OR	95% confidence interval		OR	95% confidence interval	
Total MVPA																					
None	1,558	482	1.00	Referent		1.00	Referent		n/a	n/a	n/a	105	1.00	Referent		1.00	Referent		n/a	n/a	n/a
<10 MET-hrs/wk	905	237	0.71	0.56	0.90	1.00	0.75	1.33	n/a	n/a	n/a	52	0.63	0.36	1.08	0.74	0.43	1.27	n/a	n/a	n/a
10-20 MET-hrs/wk	914	219	0.65	0.51	0.82	0.90	0.70	1.18	n/a	n/a	n/a	59	0.87	0.58	1.32	1.13	0.74	1.71	n/a	n/a	n/a
>20 MET-hrs/wk	5,360	1,148	0.55	0.46	0.65	0.99	0.82	1.19	n/a	n/a	n/a	231	0.51	0.38	0.69	0.90	0.63	1.27	n/a	n/a	n/a
*p trend*					*<0.0001*			*0.926*							*<0.0001*			*0.792*			
Leisure-time MVPA																					
None	3,925	1,160	1.00	Referent		1.00	Referent		1.00	Referent		227	1.00	Referent		1.00	Referent		1.00	Referent	
<10 MET-hrs/wk	1,165	267	0.67	0.55	0.83	0.93	0.73	1.19	0.92	0.72	1.18	68	1.01	0.70	1.46	1.15	0.79	1.66	1.15	0.79	1.65
10-20 MET-hrs/wk	1,117	216	0.51	0.40	0.64	0.73	0.58	0.92	0.72	0.57	0.90	67	0.84	0.57	1.24	1.03	0.67	1.58	1.03	0.67	1.58
>20 MET-hrs/wk	2,530	443	0.46	0.38	0.56	0.87	0.72	1.05	0.87	0.72	1.04	85	0.55	0.41	0.74	0.94	0.64	1.38	0.94	0.65	1.37
*p trend*					*<0.0001*			*0.050*			*0.038*				*<0.0001*			*0.758*			*0.756*
Occupational MVPA																					
None	4,522	1,097	1.00	Referent		1.00	Referent		1.00	Referent		239	1.00	Referent		1.00	Referent		1.00	Referent	
<10 MET-hrs/wk	570	128	0.96	0.72	1.27	1.14	0.84	1.55	1.16	0.85	1.56	38	1.21	0.70	2.10	1.29	0.72	2.30	1.28	0.72	2.27
10-20 MET-hrs/wk	540	143	1.10	0.84	1.44	1.27	0.96	1.69	1.30	0.98	1.72	32	1.11	0.67	1.82	1.13	0.71	1.80	1.13	0.71	1.79
>20 MET-hrs/wk	3,105	718	0.99	0.87	1.13	1.16	1.01	1.33	1.17	1.02	1.35	138	0.72	0.54	0.96	0.89	0.65	1.21	0.89	0.65	1.21
*p trend*					*0.970*			*0.030*			*0.018*				*0.043*			*0.509*			*0.491*

Abbreviations: MET, Metabolic Equivelent; OR, Odds Ratio. ^∗^Model 1: Unadjusted. ^†^Model 2: Adjusted for age, race/ethncity, body mass index, education, smoking status, family history of diabetes, hypertension. ^‡^Model 3: Adjusted for age, race/ethncity, body mass index, education, smoking status, family history of diabetes, hypertension, and leisure-time or occupational MVPA.

## Data Availability

The data used to support the findings of this study are available from the corresponding author upon request.
